# Attentional bias towards social interactions during viewing of naturalistic scenes

**DOI:** 10.1177/17470218221140879

**Published:** 2022-12-21

**Authors:** Simona Skripkauskaite, Ioana Mihai, Kami Koldewyn

**Affiliations:** 1 School of Psychology, Bangor University, Bangor, UK; 2 Department of Experimental Psychology, University of Oxford, Oxford, UK

**Keywords:** Social attention, social interactions, eye-tracking, adult, naturalistic scenes

## Abstract

Human visual attention is readily captured by the social information in scenes. Multiple studies have shown that social areas of interest (AOIs) such as faces and bodies attract more attention than non-social AOIs (e.g., objects or background). However, whether this attentional bias is moderated by the presence (or absence) of a social interaction remains unclear. Here, the gaze of 70 young adults was tracked during the free viewing of 60 naturalistic scenes. All photographs depicted two people, who were either interacting or not. Analyses of dwell time revealed that more attention was spent on human than background AOIs in the interactive pictures. In non-interactive pictures, however, dwell time did not differ between AOI type. In the time-to-first-fixation analysis, humans always captured attention before other elements of the scene, although this difference was slightly larger in interactive than non-interactive scenes. These findings confirm the existence of a bias towards social information in attentional capture and suggest our attention values social interactions beyond the presence of two people.

Detecting, identifying, and interpreting social cues are important for social success. Research to date shows that our attention prioritises social information above and beyond low-level physical salience (e.g., End & Gamer, 2017; Yarbus, 1967). A preference towards attending to other people allows us to quickly and accurately parse social scenes and facilitates social understanding. The interactions between people are a unique source of social cues. Not only do they provide rich and complex information about individuals, but also about the relationships *between* people (Quadflieg & Penton-Voak, 2017). Indeed, from an early age, humans use viewing social exchanges as an opportunity to learn from and about others, including assessing competence (Leetherford, 2018) and judging the likelihood of affiliation (Powell & Spelke, 2018). Watching others interacting may also provide a natural opportunity to learn social rules and norms (see Quadflieg & Koldewyn, 2017). Despite the key role interactions play in social understanding, it remains unclear whether that importance is reflected in the degree to which they capture and hold attention above and beyond the simple presence of multiple agents.

Recent evidence suggests that interacting dyads may be perceived and processed differently from either individuals or non-interacting dyads. For instance, pairs of silhouettes that face each other (i.e., interacting) are found and processed more efficiently than either dyads facing away from one another, or single individuals in a visual search task (Papeo et al., 2019). Facing dyads are also recognised as human more quickly, are processed faster and remembered better, and are more susceptible to the inversion effect than non-facing dyads (Papeo & Abassi, 2019; Papeo et al., 2017; Vestner et al., 2019). Looking time data suggests that infants are capable of differentiating between facing and non-facing dyads already at 6 months (Goupil et al., 2022) and human silhouettes facing one another also draw more visual attention in both neurotypical and autistic children (Stagg et al., 2014). Taken together, these findings suggest that attentional processing within the broader “social” category is altered by the presence of a social interaction. However, it is not yet clear whether this effect is purely social, or in part reflects a configurational benefit not specific to social stimuli (e.g., Vestner et al., 2020).

Importantly, these experiments used tightly controlled stimuli, with figures isolated from both background elements and context. Similarly, they focused on only one (important) cue to social interaction, that of facing direction. In daily life, interactions between people are encountered in any number of contexts, settings, and situations where social information competes for attention with many objects and other distractors. Indeed, one purpose of the attentional bias towards others may, at least in part, be to facilitate our ability to filter out “non-social” elements to successfully allocate attention to relevant social information (see Kingstone, 2009; Risko et al., 2012). Thus, it is crucial to investigate attention to social interaction within the kinds of complex scenes that are typically encountered in “real-life.”

To date, few studies have explicitly investigated the influence of social exchanges on how visual attention operates in complex scenes. Birmingham et al. (2008) looked at visual attentional engagement in scenes depicting either one or three individuals being either active or inactive. Three-person “active” scenes could be either interactive or not. They did not find increased attention towards humans in interactive scenes compared with active but non-interactive scenes. Although that particular analysis is only briefly described, making it unclear whether these scenes were, indeed, perceived as interactive or non-interactive. In contrast, Villani et al. (2015) found that more attention was paid to the faces and arms of humans in interactive rather than non-interactive paintings depicting two or more human figures, although they did not consider attention to other scene elements. Inclusion of some dyadic scenes in the latter, but not former, study could, however, partially explain the inconsistency in their findings. In the study by Chevallier et al. (2015), a social bias in non-autistic, and to a lesser extent autistic, children only occurred when viewing videos of two children interacting, but not when viewing dynamic or static arrays depicting two or more faces and objects. However, due to the differences in stimulus size between conditions and the authors’ focus on autistic attention, they did not report on statistical comparisons between these conditions directly and, thus, inferences can only be made based on descriptive information. Given these conflicting findings, it remains unclear whether the presence of a dyadic social interaction alters attention in complex social scenes.

The current study, thus, aimed to investigate whether the bias to attend to the social elements in naturalistic scenes is moderated by the presence of a social interaction. To address this question, we assessed looking behaviour while participants freely viewed naturalistic photographs depicting two people who were either interacting with each other, or not. Based on the overwhelming previous evidence for a social attentional bias, we expected that humans in the scenes would receive more attentional engagement (i.e., longer dwell time) and faster attentional capture (i.e., shorter time-to-first-fixation) than other elements in the scene (Hypothesis 1). We also hypothesised a stronger social bias via longer engagement with and faster capture towards humans in scenes judged as depicting two interacting than two non-interacting individuals (Hypothesis 2). These data should give us a clear insight into whether the third-person observation of interacting individuals carries important social information over and above the observation of a non-interacting pair.

## Methods

### Participants

An a priori power analysis determined that a sample size of 70 (β = 0.80, α = .05) would be sufficient to detect a large effect size (Cohen’s *f* = .40) in the three-way interaction (G*Power 3.1; Faul et al., 2009). This was pre-registered on AsPredicted (https://aspredicted.org/vf8rr.pdf). In total, 73 participants were recruited from the general population through an opportunity sample. Data from two participants who were outside our target age-range, and one who was falling asleep were removed. The final sample of 70 participants (age: *M* = 21.07, *SD* = 2.63, range: 18–34; 47 females and 1 other) had normal or corrected-to-normal vision. Participants were excluded if they did not have normal or corrected-to-normal vision, but were not screened or assessed for any neurodevelopmental or mental health conditions. All participants gave informed consent and were reimbursed for their time in the study with course credit or a small monetary reward. The study was approved by the School of Psychology’s ethics board and was conducted in accordance with the 1964 Helsinki declaration.

### Stimuli and apparatus

**Figure 1.**
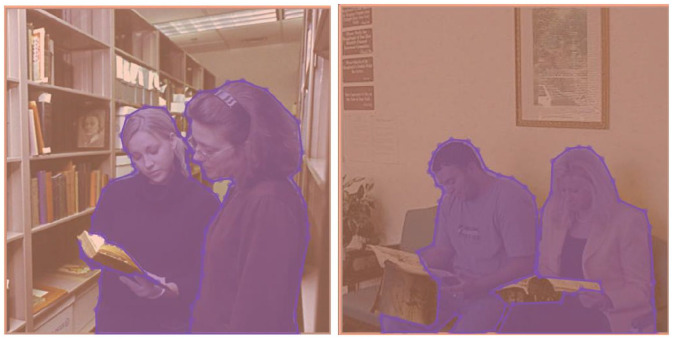
Example stimuli and AOI definition in interactive (left) and non-interactive (right) condition. Definition of areas of interest (AOI): social (blue/darker grey) and non-social (orange/lighter grey). Images adapted from the SUN database (Xiao et al., 2010).

Stimuli were presented in a paradigm coded in PsychoPy 2 (Peirce et al., 2019), while data were collected with a laptop-mounted Eyelink Portable Duo eye-tracker (SR Research Ltd, 2012). Stimuli were presented on a 380 × 215 mm^2^ (1920 × 1080 px) monitor with a grey background. Participants’ binocular gaze^1^ was tracked remotely at a sampling rate of 1000 Hz. Target stimuli were 60 photographs selected from the SUN database (Xiao et al., 2010). All the stimuli in this study depicted two people in naturalistic scenes (see Supplementary materials: Figures S1 and S2). They did not include any photographs with direct gaze at the camera, interactions with unseen (off-camera) agents, or obvious depictions of strong emotions. Initially, 127 pictures depicting two people in everyday context (i.e., shops, offices, playgrounds, etc.) were chosen and then rated for their interactiveness (1 = *Not at all interactive*, 7 = *Very interactive*) by 26 independent judges (age: *M* = 24.77, *SD* = 4.03, range: 21–33; 16 females) recruited via opportunity sampling from the same population as study participants. Based on these ratings, the 30 photographs that received the lowest (*M* = 1.86, *SD* = 0.40) and the 30 that received the highest (*M* = 5.40, *SD* = 0.54) average scores were selected to represent “non-interactive” and “interactive” categories (Figure 1), respectively. All images were pre-processed in Photoshop graphic software (version CC 2019) by removing colour cast and matching the colour scheme to one of the images (using the “match colour” function) and at a standard size of 860 × 860 px (13.6° × 13.6°). For the purposes of the analysis, each photograph was further divided into areas representing social and non-social aspects of the image (see Figure 1 and section “Data analysis”).

### Procedure

The full experiment consisted of 142 trials in total: 60 trials specific for this experiment and 82 images for another paradigm.^2^ Data from other images will not be presented here. Data collection took place in a custom designed eye-tracking lab (outfitted with light blocking blinds) with participants sitting at a height-adjustable table and facing a plain wall while wearing noise cancelling headphones. One or two trained operators were present in the room, but were out of participants’ sight, during the data collection. Before the experiment, participants received a verbal explanation and demonstration of how the eye-tracker works and how head movements can impact data quality. Each participant then went through an experimenter-operated 13-point (black-on-grey) calibration and validation procedure. Automatic calibration was attempted first, but multiple manual calibration procedures were carried out, if needed, till good calibration and validation were achieved. Each participant received on-screen and verbal instructions to simply look at the photographs presented on the screen. Every trial first started with a drift correction dot at the centre of the screen, which remained until participants pressed the “space” button while successfully focusing on the dot. The image for that trial was then presented on the left or right side of the screen for 5,000 ms. The leftmost edge of the right-hand pictures, and the rightmost edge of the left-hand pictures were placed 1° from the centre. Thus, the image never overlapped with the centre of the screen. Whether a picture appeared on the right or the left was counterbalanced between participants and the order of trials was fully randomised for each participant. Participants were presented with an option to take a self-paced break (without leaving the desk) every 28 trials.

### Data analysis

For each photograph, social and non-social areas of interest (AOI; Figure 1) were defined using the “freehand” function in Eyelink Data Viewer (SR Research Ltd, 2013). The former encompassed all visible clothed and unclothed body parts (including faces/heads) that were not out of the frame and not obstructed by other objects, whereas the latter included everything else in the scene, including background elements (c.f. Fletcher-Watson et al., 2009). This resulted in interactive images consisting of, on average, 34% social AOI (*M* = 259380.00 px, *SD* = 104248.80) and 66% non-social AOI (*M* = 505413.40 px, *SD* = 104861.70), whereas non-interactive images consisted of on average 26% of social AOI (*M* = 199356.00 px, *SD* = 127112.60) and 74% non-social AOI (*M* = 565634.10 px, *SD* *=* 127570.80). While social AOIs were smaller than non-social AOIs in both interactive, *t*(116) = −12.18, *p* < .001, *d* = −1.13, and non-interactive, *t*(116) = −8.18, *p* < .001, *d* = −0.76, conditions, there were no significant differences between the interactive and non-interactive conditions when comparing the size of social, *t*(116) = 2.00, *p* = .146, *d* = 0.19, or non-social, *t*(116) = −2.00, *p* = .144, *d* = −0.19, AOIs (see Supplementary Materials: Tables S1–S3 for full comparison of AOI sizes). Dwell time, the amount of time spent fixating inside that particular AOI (SR Research Ltd, 2013), was extracted for both social and non-social AOIs as a measure of general attentional engagement with that AOI. As the images never appeared at the same place as the drift correction dot, time-to-first-fixation to social and non-social AOIs was extracted as a measure of attentional capture.

To avoid the data distortion due to blinks or poor signal (c.f. Fletcher-Watson et al., 2009), all trials where less than 33% of viewing time was engaged with the target photograph were treated as missing. This included both time when participants might be blinking, saccading between fixations, looking off-screen, and times when data might be missing due to technical issues. This did not, however, result in the loss of very much data. This procedure resulted in the average exclusion of 0.2% (*M* = 0.14, *SD* = 0.39, range: 0–2) of trials per participant. On average, participants fixated on the screen for 75.3% of the time (*SD* = 4.50, range: 38.8%–87.1%) of included trials.

Two different models were then used to assess general attentional engagement and attentional capture, respectively. Dwell time and time-to-first-fixation were analysed separately using linear mixed-effect modelling with a 2 (condition: interactive and non-interactive) × 2 (AOI: social and non-social) design (lme4 package; Bates et al., 2015). Satterthwaite’s approximation method was utilised to enable significance testing (lmerTest package; Kuznetsova et al., 2017). A crossed random effect structure was defined, where dwell times or time-to-first-fixations were nested within two random factor structures: within individuals and within stimuli, separately. AOI information with AOI type (social or non-social) as predictors was further nested under the stimulus information. Post hoc Tukey’s honestly significant difference (HSD) pairwise comparisons were carried out when applicable (emmeans package; Lenth, 2018).

## Results

### Attentional engagement

**Figure 2.**
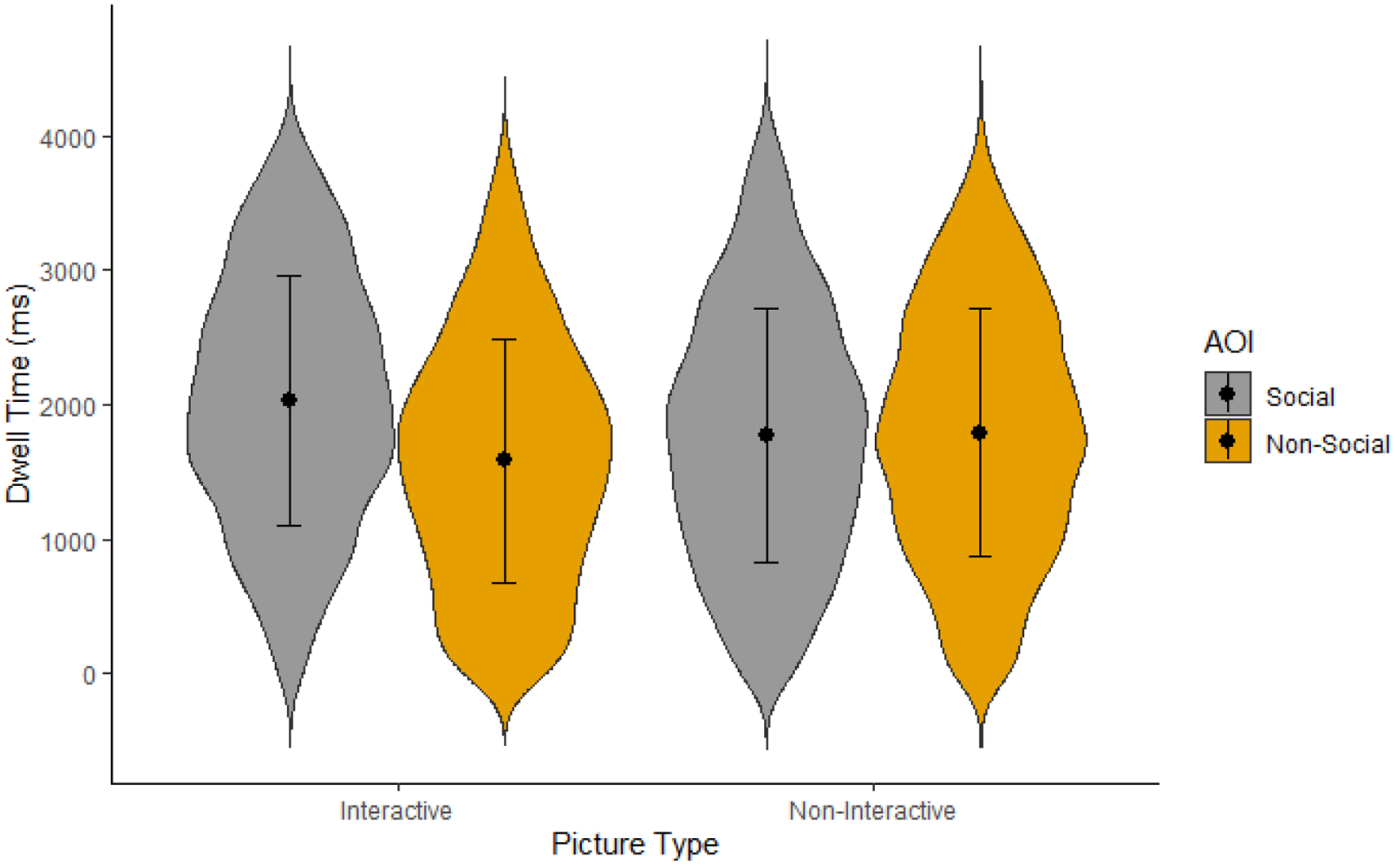
Violin plot for participants’ mean dwell time per AOI and scene type. Error bars represent standard deviations.

Dwell time was examined to determine whether the amount of attention paid to social information, in contrast to the rest of the image, differed based on whether the scene was interactive or not. Results showed that the main effect of scene type did not reach significance, *F*(1, 120) = 0.06, *p* *=* .809, η^2^_p_ < .01, confirming that looking time did not differ based on whether the scene was interactive (*M* = 1,804.73, *SD* = 920.09) or non-interactive (*M* = 1,782.92, *SD* = 928.70) when averaged across social and non-social AOIs. There was, however, a significant main effect of AOI type, *F*(1, 120) = 4.19, *p* *=* .043, η^2^_p_ = .03. Participants on average looked at social information (*M* = 1,866.17, *SD* = 932.92) more than non-social information (*M* = 1,721.49, *SD* = 910.20) across scenes. This effect was moderated by the type of scene (Figure 2), *F*(1, 120) = 5.06, *p* *=* .026, η^2^_p_ = .04. Specifically, participants looked at social (*M* = 1,986.53, *SD* = 910.75) more than non-social (*M* = 1,622.93, *SD* = 893.27) AOIs in the interactive scenes, *t*(62.1) = 2.97, *p* *=* .004, *d* = 0.38. Yet, in the non-interactive scenes they looked just as long at both social (*M* = 1,745.58, *SD* = 939.49) and non-social AOIs (*M* = 1,820.25, *SD* = 916.49), *t*(62.1) = −0.14, *p* *=* .889, *d* = −0.02. Indeed, participants numerically looked at non-social elements for longer than social elements in non-interactive scenes, though this difference is too small for us to consider it interpretable.

### Attentional capture

Participants’ time-to-first-fixation on social and non-social AOIs were assessed to evaluate potential differences in attentional capture between interactive and non-interactive scenes. To correct for normality violations, log-transformed data was used in the analysis. Participants were on average missing attention capture data on 1% (*M* = 0.30, *SD* = 0.55) of social AOIs and 2.77% (*M* = 0.83, *SD* = 1.14) of non-social AOIs for interactive pictures, *t*(69) = 3.45, *p* = .001. For non-interactive pictures, they were missing 2.37% (*M* = 0.71, *SD* = 0.92) of data for social AOIs and 2.43% (*M* = 0.73, *SD* = 1.02) for non-social AOIs, *t*(69) = 0.09, *p* = .927. For trials with data missing on one AOI, only the data for the available AOI were modelled.

**Figure 3.**
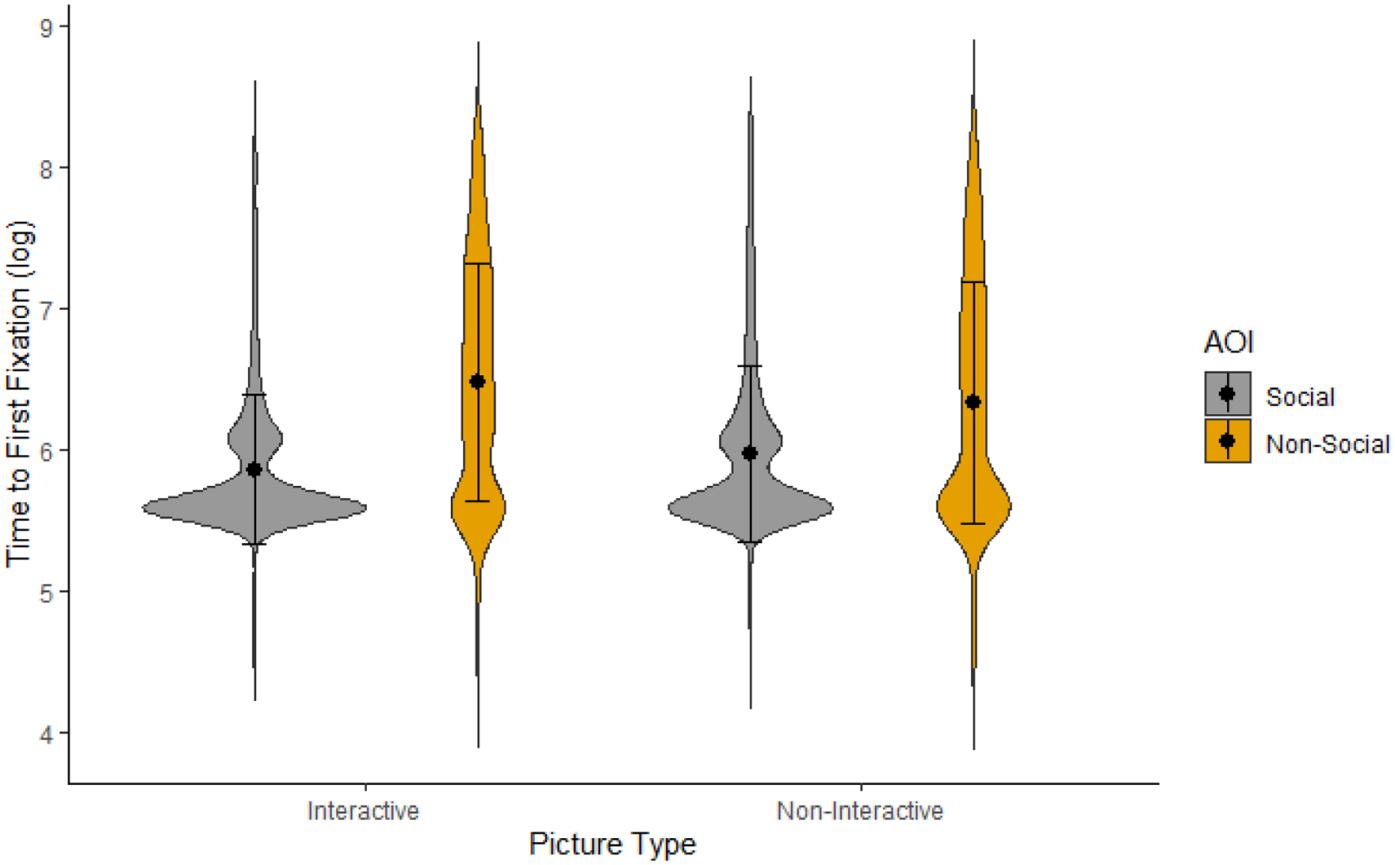
Violin plot for participants’ mean log-transformed time-to-first-fixation per AOI and scene type. Error bars represent standard deviations.

Unsurprisingly, multilevel analysis showed that time-to-first-fixation to the scene overall did not differ based on whether scenes were interactive (*M* = 673.33, *SD* = 713.73) or non-interactive (*M* = 676.40, *SD* = 724.94), *F* (1, 119.26) = 0.02, *p* = .888, η^2^_p_ < .01. Similar to dwell time analyses, there was both a significant main effect of AOI type, social: *M* = 483.98, *SD* = 535.80; non-social: *M* = 867.52, *SD* = 822.22, *F* (1, 119.26)= 60.46, *p* < .001, η^2^_p_ = .34, and an interaction effect between AOI and scene type (Figure 3), *F* (1, 119.26) = 4.65, *p* = .033, η^2^_p_ = .04. In interactive scenes, participants looked at social AOIs (*M* = 440.54, *SD* = 462.51) faster than at non-social AOIs (*M* = 910.34, *SD* = 836.14), *t*(62) = −6.91, *p* < .001, *d* = −0.88. To a lesser extent, their attention was also captured more quickly by social (*M* = 528.11, *SD* = 598.08) than non-social (*M* = 824.77, *SD* = 806.03) AOIs in non-interactive scenes, *t*(138) = −3.91, *p* < .001, *d* = −0.50.

## Discussion

While much prior research has confirmed the “social attention bias,” we demonstrate here that this bias is moderated by the presence (or absence) of social interactions. The currents findings are partially in line with previous research on both social and non-social attention in non-clinical adult samples (e.g., Bindemann et al., 2005, 2010; Fletcher-Watson et al., 2008; Rigby et al., 2016). Indeed, when interactions are not taken into account, our results confirmed previous findings that people orient faster to humans and look at them for longer than other elements in a scene. However, our findings also reveal that this social bias in attentional engagement only occurred when people in the scene were involved in a social exchange. In non-interactive scenes, participants explored the background for just as long as the people. In contrast, participants were faster to orient to human information in *both* interactive and, to a lesser degree, non-interactive scenes.

We hypothesised a stronger social bias towards humans in interactive than non-interacting scenes but, given the overwhelming previous evidence for an attentional bias to human information more generally, we did not expect that this social bias would occur only in interactive scenes. Most previous research on social and non-social attention has not directly compared interactive and non-interactive scenes, but instead has looked at attention to isolated individual faces (Bindemann et al., 2005), single person scenes (Bindemann et al., 2010; Fletcher-Watson et al., 2008), or mostly interactive scenes only (e.g., Klin et al., 2002; Rigby et al., 2016; Riby & Hancock, 2008). Therefore, it is difficult to say how our finding of a lack of social bias in attentional engagement towards humans in non-interactive scenes compares with previous research investigating qualitatively different social stimuli. Indeed, those few studies that have previously explicitly investigated the effect of social interactions on looking behaviour provide inconsistent results (Birmingham et al., 2008; Villani et al., 2015). Our findings are in line with those of Villani et al. (2015), who also found that people looked more at human information in interactive paintings than in non-interactive paintings, but did not compare social versus non-social attention within images. They are also in line with the findings of Chevallier et al. (2015), showing that attentional social bias in children only occurred when viewing interactive rather than non-interactive stimuli. However, our findings partially contradict those of Birmingham et al. (2008), who did not observe differences between interactive and individual-action scenes depicting three people. Stimulus and design differences between our study and that of Birmingham et al. (2008) could explain these divergent results. For example, it is possible that an individual may be perceived as more unusual and thus may capture attention more quickly and be processed for longer than two non-interacting individuals; while the presence of additional people in a scene may change attention to interactions themselves. It is also possible that viewing both interactive and non-interactive images within the same task biases participants to focus more specifically on the differences between the scenes (interactiveness in our study and action in Birmingham et al., 2008).

The current findings are, however, in line with previous research using less naturalistic stimuli (Papeo & Abassi, 2019; Papeo et al., 2017, 2019; Stagg et al., 2014; Vestner et al., 2019). While previous research shows that interactions (i.e., facing figures) are processed differently than non-interactions (i.e., non-facing figures), the current research expands on previous findings by showing that interactions are also processed differently when encompassing interactive cues other than facing direction, which better reflects interactions encountered in “real-life.” It should be noted, however, that to control for the attention biasing effects of motion (see Thompson & Parasuraman, 2012) between social and non-social AOI, the current study utilised only static rather than dynamic stimuli. Future research is needed to confirm that this social interaction bias remains in even more naturalistic scenes involving additional cue of dynamic action. Nevertheless, in our view, taken together these findings further support the notion that the human perceptual system may be at least partially tuned to dyadic social interactions beyond the presence of two people.

Several underlying mechanisms may be responsible for this effect of increased attention to social interactions. First, interactive scenes may carry additional social value and thus increased richness and meaning compared with non-interactive scenes. After all, in addition to providing information about separate individuals, interactions may also provide information about people’s interpersonal relationships (see Quadflieg & Koldewyn, 2017; Quadflieg & Penton-Voak, 2017). Second, multiple visuospatial properties known to guide visual attention are likely to be more pronounced in interactive scenes. In particular, interactors are more likely to face towards each other than non-interactors and the facing direction of human bodies and the gaze direction of human eyes both act as directional attention cues. Prior research has shown that both humans and objects that direct attention (e.g., arrows, lamps) are more effective distractors and are found more quickly when facing each other than in non-facing arrangements (i.e., facing away or facing the same direction) in a visual search task (Vestner et al., 2020, 2022). Thus, it is possible that previous research showing faster and more efficient processing of pairs of figures facing one another as opposed to figures facing away or the same direction reflects a simple configurational benefit rather than an increased social bias towards observed social interactions. This “attentional cuing effect” likely contributes to the faster attentional capture by interacting agents seen in the current study. After all, while none of the interactive scenes in the current study depicted individuals facing away from one another, at least 7 of the 30 non-interactive scenes did (see Supplementary materials: Figure S1 and S2). However, the cuing effect is less likely to be a significant contributor to greater engagement with interacting than non-interacting humans. In addition, the set of interactive scenes used in the current study are quite diverse and only about one-third of them show dyads that are directly facing each other. Many scenes contain other cues to social interaction, particularly joint attention to an object in the scene where the attention cuing effect should drive attentional capture by the object rather than the people themselves. Thus, while attentional/directional cues within the scenes likely contribute to our attentional capture effects, we think it unlikely that attentional cuing can explain the full set of results.

There are several other visuospatial properties in scenes that will tend to differ between interactive and non-interactive scenes, including not only facing direction but also interpersonal distance, body postures, and alignment between actions and/or gaze direction. Although interpersonal distances in the current stimulus set were roughly similar (evaluated by pixel distances between individuals’ centre-of-mass), it is likely that the interactive and non-interactive scenes in the current study differed from each other on a number of dimensions. That is not surprising, given that closer interpersonal distances, more direct interpersonal angles, and more open postures lead to a higher probability of social scenes being judged as interactive (Zhou et al., 2019) and scenes in the current study were categorised as interactive, or not, based on the evaluation of independent judges. Similarly, the differences in social AOI size between interactive and non-interactive images in the current stimulus set were not statistically significant and were accounted for in the analytical design. However, they were not exactly matched between conditions, likely at least partially due to non-interactive scenes more often depicting humans on different planes. A larger proportion of pictures depicting children were included in the interactive pictures than in the non-interactive scenes. Whether this might be a result of scenes with children being generally characterised as more interactive than non-interactive is difficult to determine. Most scenes depicting children in the original set (30 out of 43) were not consistently classified as either. However, how social information is positioned in space, its size, and differing agent characteristics, including age, could be partially responsible for the observed differences in attentional processing between interactive and non-interactive scenes. It has to be noted that the aim of the current study was to determine whether perception of interaction (as a combination of different cues that lead to it being perceived as such) relates to faster orienting and prolonged attention beyond the established social attentional bias. A much larger stimulus set would need to be used to reliably disentangle individual effects of such interactive cues as facing direction, mutual eye-gaze, joint attention, positioning, and size across interactive and non-interactive scenes.

Our findings further our understanding of social scene perception by suggesting that the presence of a social interaction greatly strengthens the social bias as indexed by attentional engagement. We found that the presence of a social interaction fully moderates attentional engagement to social versus non-social elements of naturalistic scenes. At the same time, this “interaction effect” was not as strong for attentional capture. Although the bias to look first at humans was stronger in scenes that contained a social interaction, participants looked at humans faster than at other elements of the scene whether scenes were interactive or not. Taken together, these findings offer insight into a potential hierarchy of attentional processes that occur when viewing social scenes. Specifically, it lends strength to the idea that the social bias is an automatic bottom-up process that guides our attention, even in cluttered scenes. This is in line with other studies showing faster orienting to human than other information within scenes (Bindemann et al., 2010), quicker fixations on scenes with humans over concurrently presented non-social scenes (Fletcher-Watson et al., 2008), and that social features partially override the impact of low-level physical salience during scene perception (Rösler et al., 2017). Our results further suggest that the informative value of social interactions may guide one’s attention during initial rapid orienting, but more strongly influences attention during further exploration, once the locations of relevant scene elements have been determined. It is indeed intuitive that we would look at any social information faster than non-social information, but only continue to stay engaged with information of particular relevance. However, as the current study only compared the existence of a social bias between trials in different conditions, only tentative conclusions can be drawn on prioritisation of orienting towards interacting over non-interacting individuals who are present in the same scene. Future studies involving within trial/scene comparisons are necessary to further disentangle these processes, including investigating scenes that introduce additional agents into the scene, which may lead to changes in attention and processing of social interactions. Furthermore, as the current study focused on participants from the general adult population, future research should investigate whether currently observed social interaction bias applies to other groups that are known to process social information differently including younger (e.g., Doherty et al., 2019) or autistic (e.g., Chevallier et al., 2015) individuals.

## Supplemental Material

sj-docx-1-qjp-10.1177_17470218221140879 – Supplemental material for Attentional bias towards social interactions during viewing of naturalistic scenes Click here for additional data file. Supplemental material, sj-docx-1-qjp-10.1177_17470218221140879 for Attentional bias towards social interactions during viewing of naturalistic scenes by Simona Skripkauskaite, Ioana Mihai and Kami Koldewyn in Quarterly Journal of Experimental Psychology

## Notes

1 Monocular gaze data, chosen on the basis of more accurate calibration, was used in the analysis.

2 These additional images were similar to target stimuli in that they were photographs of people in naturalistic scenes, but they depicted more than two people or were not unambiguously interactive/non-interactive.
