# Supplementary material for: Attentional bias towards social interactions during viewing of naturalistic scenes
Source: Q J Exp Psychol (Hove). 2022 Dec 21;76(10):2303–11. doi: 10.1177/17470218221140879 (PMC10503253; doi:10.1177/17470218221140879)
Supplement: sj-docx-1-qjp-10.1177_17470218221140879 – Supplemental material for Attentional bias towards social interactions during viewing of naturalistic scenes [file sj-docx-1-qjp-10.1177_17470218221140879.docx]

Supplementary Materials

**Figure S1**

*Stimuli set used in the interactive condition*


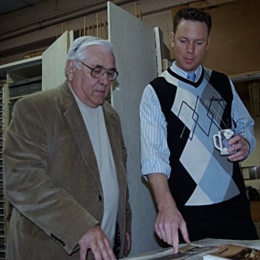

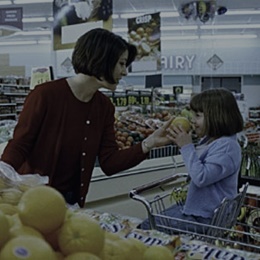

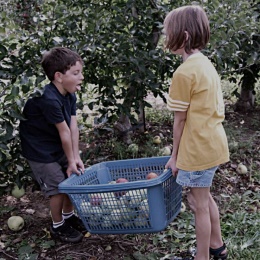

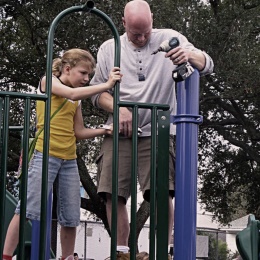

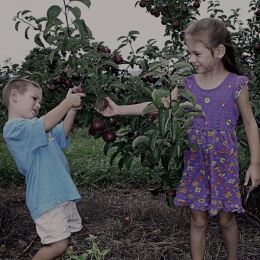

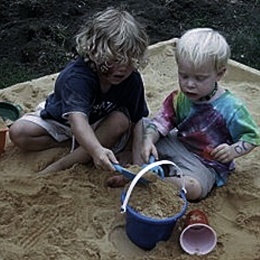

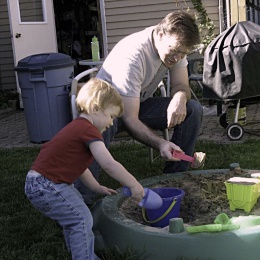

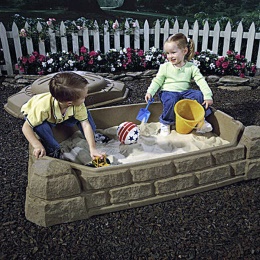

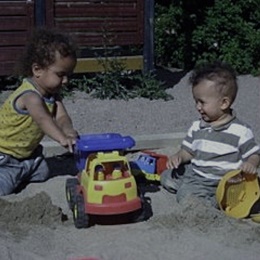

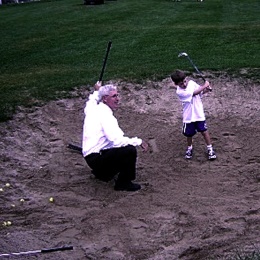

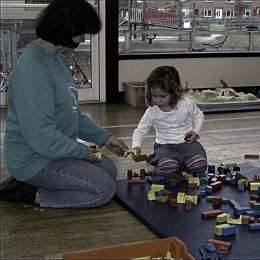

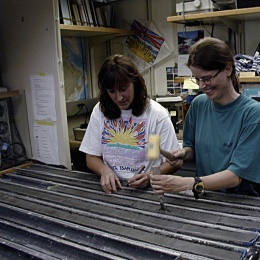

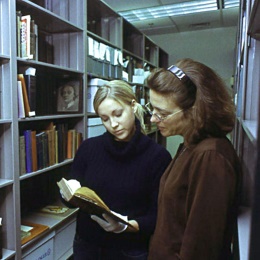

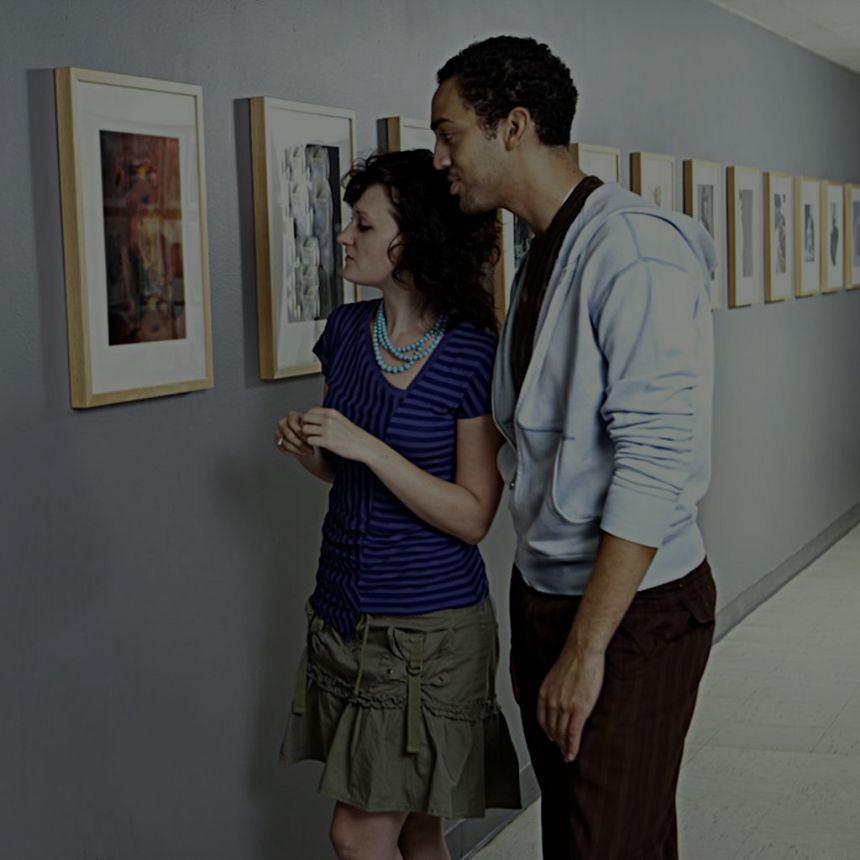

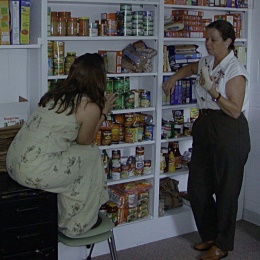

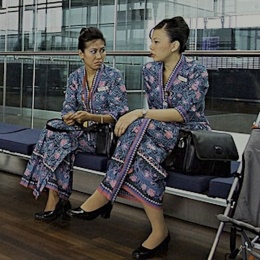

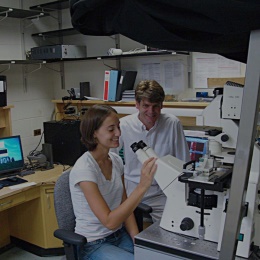

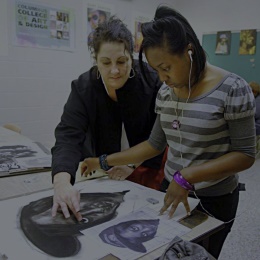

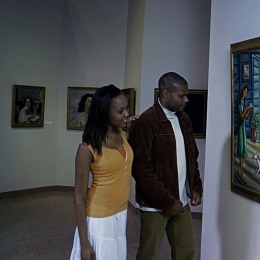

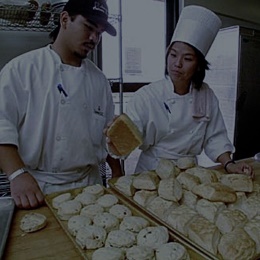

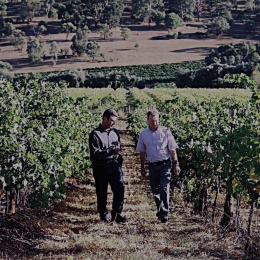

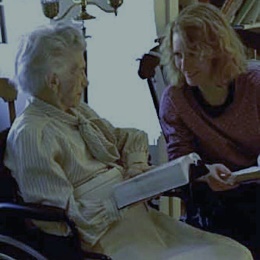

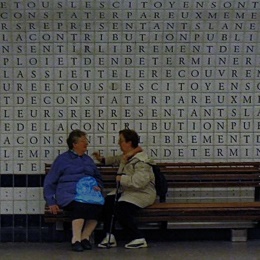

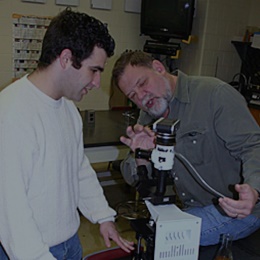

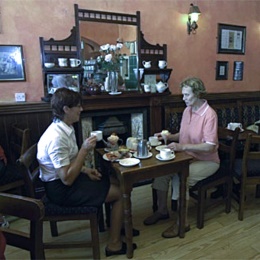

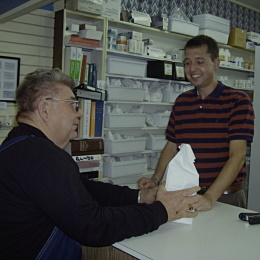

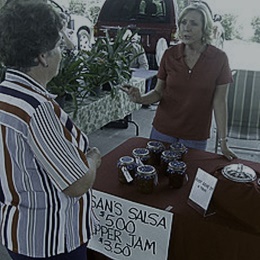

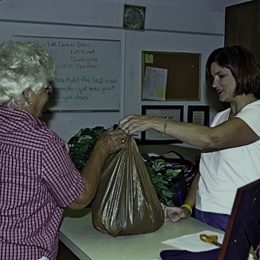

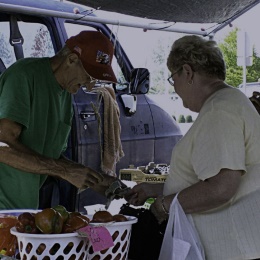

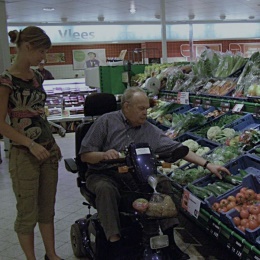


Note. Images were taken from the SUN database (Xiao et al., 2010).

**Figure S2**

*Stimuli set used in the non-interactive condition*


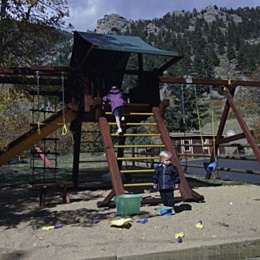

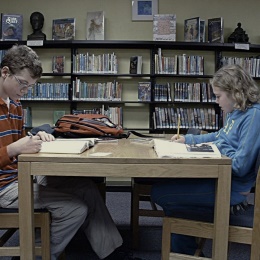

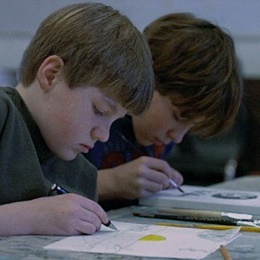

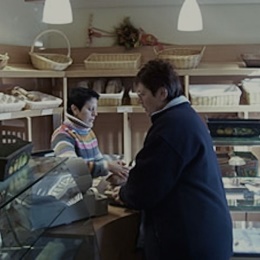

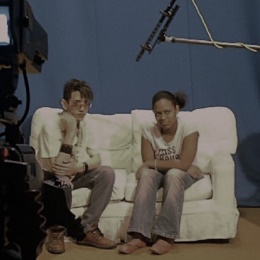

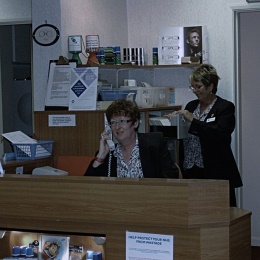

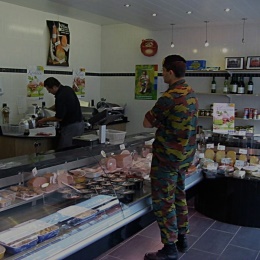

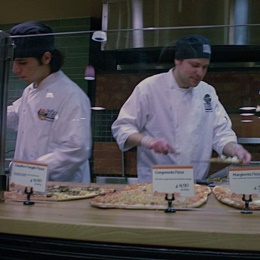

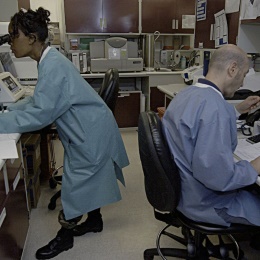

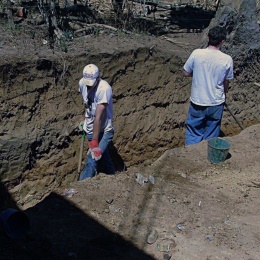

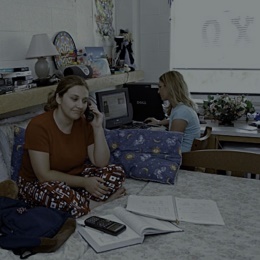

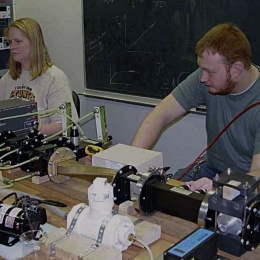

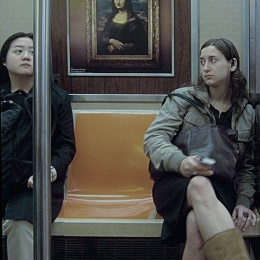

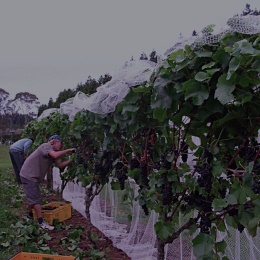

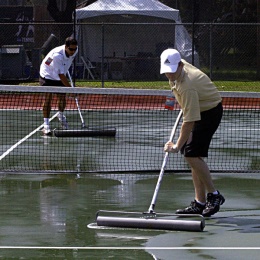

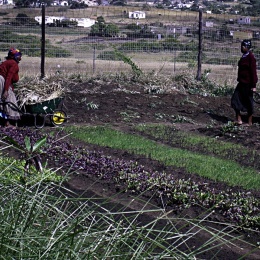

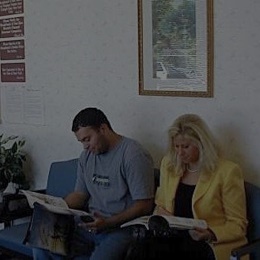

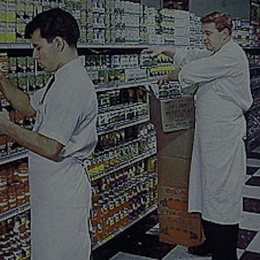

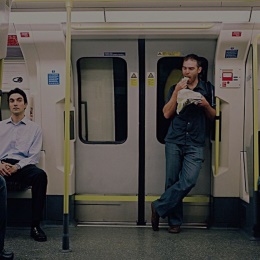

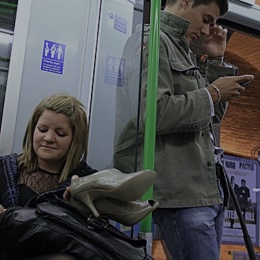

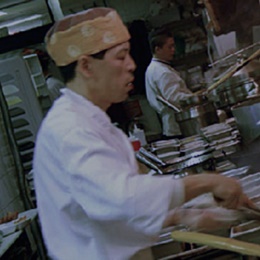

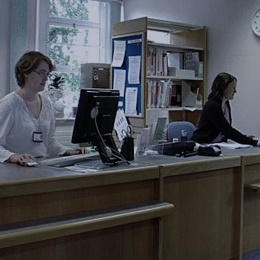

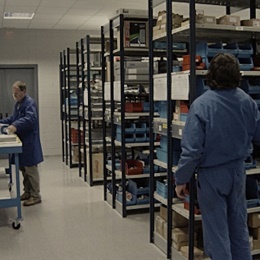

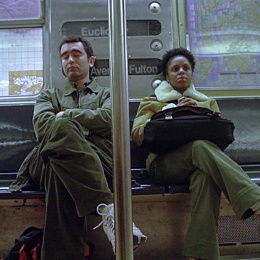

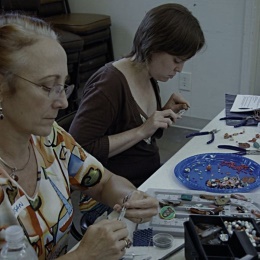

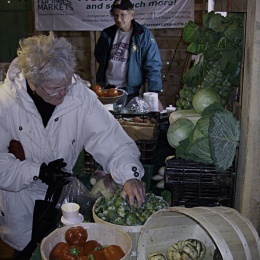

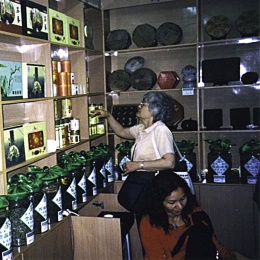

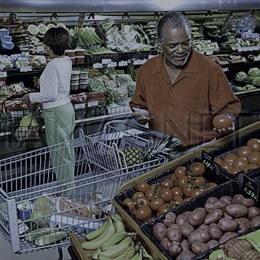

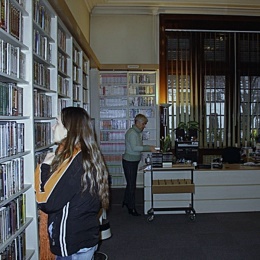

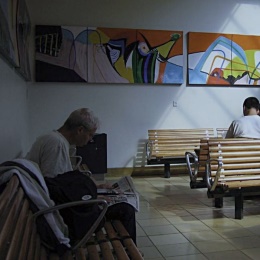


Note. Images were taken from the SUN database (Xiao et al., 2010).

**Table S1**

*Descriptive statistics for AOI sizes across conditions*

| Type of scene | AOI | *Mean* (px) | *SD* |
| --- | --- | --- | --- |
| Interactive | |  |  |
|  | Social | 259380.00 | 104248.80 |
|  | Background | 505413.40 | 104861.70 |
| Non-interactive | |  |  |
|  | Social | 199356.00 | 127112.60 |
|  | Background | 565634.10 | 127570.80 |

**Table S2**

*Main effects and interactions for a 2 (type of scene) x 2 (type of AOI) mixed ANOVA on the AOI size in pixels*

|  | *F*(1,116) | *p* | η^2^_p_ |
| --- | --- | --- | --- |
| Type of scene | 0.00 | .996 | < .001 |
| AOI | 207.16 | < .001 | .64 |
| scene * AOI | 7.99 | .006 | .06 |

**Table S3**

*Pairwise comparisons for the scene*AOI interaction*

| Contrast | | *t*(116) | *p* | *d* |
| --- | --- | --- | --- | --- |
| Social - Background | |  |  |  |
|  | Interactive scenes | - 8.18 | < .001 | - 0.76 |
|  | Non-Interactive scenes | - 12.18 | < .001 | - 1.13 |
| Interactive – Non-interactive | |  |  |  |
|  | Social AOI | 2.00 | .146 | 0.19 |
|  | Background | - 2.00 | .144 | - 0.19 |

 Note. Corrected with HSD Tukey for multiple comparisons
